# Nutrition intervention in the management of novel coronavirus pneumonia patients

**DOI:** 10.1515/biol-2022-0015

**Published:** 2022-03-22

**Authors:** Haina Cai, Yang Wang, Zejun Cai, Yuqing Lin, Qinghong Xu

**Affiliations:** Department of Nursing, Ningbo First Hospital, 59 Liu Ting Street, Haishu District, Ningbo, 315010, Zhengjiang, China; Department of Thoracic Surgery, Ningbo First Hospital, Ningbo, 315010, Zhengjiang, China; Department of Gastrointestinal Surgery, Ningbo First Hospital, Ningbo, 315010, Zhengjiang, China

**Keywords:** novel coronavirus pneumonia, nutrition intervention, nutrition management

## Abstract

In this study, we explored the effect of nutrition intervention on the management of patients with novel coronavirus pneumonia (NCP). A total of 28 NCP patients receiving therapy in Ningbo First Hospital (China) were enrolled in this study. The nutrition risk was assessed using the Nutritional Risk Screening (NRS)-2002 for the patients subjected to nutrition intervention provided by the Nutritional Department in Ningbo First Hospital, China. Compared to the situation at admission, the body mass index (BMI) and weight of NCP patients were higher at the time of discharge, while no significant difference was observed (*P* > 0.05). The serum albumin and hemoglobin levels of NCP patients were significantly increased compared with those at admission and one week after admission (*P* < 0.05). The nutrition intervention can improve the nutritional status and prognosis of NCP patients.

## 1 Introduction

Novel coronavirus pneumonia (NCP), also termed as severe acute respiratory syndrome coronavirus 2 (SARS-CoV-2), typically refers to an acute infectious pneumonia disease [1,2,3,4]. NCP has been spreading worldwide, especially in America, India, and Brazil, and the dramatically increased number of infected patients poses additional difficulty for the medical system [5,6,7,8]. NCP turns out to be characterized by quite broad clinical ranges, including asymptomatic infection, mild upper respiratory infection, severe pneumonia accompanied with respiratory failure, as well as multiple organ failure [9,10]. The main symptoms include fever, dry cough, fatigue, myalgia, and breathing difficulty. Many infected cases are subjected to hospitalization for intensive nursing. At this stage, the dominant medical care programs consist dominantly of symptomatic treatment and prevention of secondary infection [11,12], while the effective anti-virus treatment against NCP remains to be explored. Although some antivirus drugs (like oseltamivir, ganciclovir, ribavirin, etc.) have been studied in terms of their potential effect in preventing respiratory complications, their therapeutic effect in the clinic, however, awaits further verification [13,14,15].

It should be noted that nutrition intervention has always been part of the therapy protocols for acute and chronic diseases [16,17], which finds particular availability for the treatment of diseases with unknown pathogenesis. For example, the Ebola virus in West Africa (2014–2016) [18] indicated that supportive nursing helps to dramatically decrease the mortality rate. This may also work for the current NCP. New evidence implies that NCP is related to elderly patients with various complications and hypoalbuminemia [19]. Although the circulating level of albumin is not expected to serve as the nutrition marker for a patient’s inflammatory response, a recent study claimed that the low albumin level could evolve into acute respiratory distress syndrome [20]. The combined findings point to the possibility that malnutrition may exert an important impact on the final results. The nutrition status may be a potential factor influencing the prognosis of NCP patients, while the effect of early-stage nutrition support on the infected patients remains largely unexplored.

The protocols obtained from clinical practice during the treatment of NCP patients may stimulate the research on the nutrition supply scheme, which can further help to determine the standardized nutrition methods and optimum nursing procedures [21]. Here, we summarized the experience in nutrition intervention during NCP treatment to provide references for nutrition support as well as epidemic prevention and control.

## 2 Methods

### 2.1 Subjects

A total of 28 NCP patients in the isolation wards of Ningbo First Hospital during February 2–14, 2020 were enrolled. Inclusion criteria were as follows: patients were diagnosed as NCP according to the standards in Diagnosis and Treatment of New Coronavirus Pneumonia (Trial Fifth Revised Edition); patients with historical epidemiology and symptoms of fever and/or upper respiratory infection; and had positive results of the nucleic acid test of NCP by throat swab (the patients with positive results of nucleic acid test during preliminary screening in Ningbo First Hospital, China, were subsequently subjected for double check by Ningbo Center for Disease Control and Prevention, China). Exclusion criteria were as follows: NCP patients in severe and critical conditions and those who required tracheal intubation and/or suffered from gastrointestinal dysfunction. Finally, 28 NCP patients (male: 10, female: 18) with an average age of 55.79 ± 14.53 years and a BMI index of 24.43 ± 3.19 kg/m^2^ were enrolled.


**Informed consent:** Informed consent has been obtained from all individuals included in this study.
**Ethical approval:** The research related to human use has been complied with all the relevant national regulations, institutional policies and in accordance with the tenets of the Helsinki Declaration, and has been approved by the ethics committee of Ningbo First Hospital, China (Approval No.: 2020-R105).

### 2.2 Nutritional assessment

The nutrition risk of patients was assessed using the Nutritional Risk Screening (NRS)-2002 at admission and then evaluated once a week. Trained medical graduates carried out anthropometric measuring. The height and weight profiles were recorded for the patients with bare feet and light clothing. All patients were weighed once a week to calculate the body mass index (BMI). The venous blood was collected for the patients with empty stomachs in the morning to determine the hemoglobin and serum albumin levels using the automatic biochemical analyzer (Siemens, ADVIA 2400). These indicators were recorded for the patients at admission, one week after admission, and before discharge.

### 2.3 Nutrition intervention methods

It is generally acknowledged that the disease-resistance capability of patients can be improved by nutrition intervention. The nurses conducted propaganda and education of healthy diet for enrolled patients upon their admission, who also distributed daily recipes formulated by the Nutrition Department and highlighted the importance of diet for disease rehabilitation.

The food consumption of patients and related energy-level analyses were carried out by nursing staff. Particular guidance for the patients who fell below the diet standards was also conducted to emphasize the importance. The nutrition and energy levels were also made clear to these patients upon distributing the food.

For the NCP patients with the absence of nutritional risk, nutrition intervention was performed based on “Nutritional Dietary Guidelines for NCP Prevention and Control” to formulate dietary plans. The daily recipe consisted of 250–400 g cereals, 150–200 g high-quality proteins, >300 g milk or dairy products, >500 g vegetables, and 200–350 g fruits. The recipe in more detail varied slightly among different individuals with different tastes. Specifically, the breakfast mainly included one egg, 250 mL milk, one Chinese steamed bun (randomly provided with meat, vegetable, and multigrain farci), and 200 g porridge. The brunch (provided at 10:00 a.m.) consisted of 150 g orange. The lunch included 200 g rice, 300 g vegetable, 100 g meat, and 50 g shrimp. Apple or pear (150 g) was provided at 3:00 p.m. The dinner consisted of 200 g rice, 300 g vegetable, 100 g fish, and 100 g bean products. Yogurt (200 mL) was provided at 9:00 p.m. Besides, daily water consumption was 2000 mL. For the NCP patients with nutritional risk, the recipe was additionally given 200 mL milk (half-hour after lunch) and 200 g steamed egg custard (lunch) to increase the intake of high-quality proteins.

Patients were required to do exercise three times per day (15–20 min each time) to increase the peristalsis of the gastrointestinal tract.

### 2.4 BMI and serum index

BMI was calculated based on the measured weight and height parameters [22]. The criteria for malnutrition is as follows: (1) BMI <16.0, severe protein-energy malnutrition; (2) BMI = 16.0–16.9, moderate protein-energy malnutrition; (3) BMI = 17.0–18.4, mild protein-energy malnutrition; (4) BMI = 18.5–23.9, normal; (5) BMI ≥24.0, overweight; and (6) BMI ≥28.0, obesity.

### 2.5 Statistical analysis

SPSS 21.0 software was utilized to carry out statistical analysis. The measurement data followed normal distribution were displayed as mean ± standard deviation (SD) and analyzed by *t*-test for two independent samples. The enumeration data were expressed as rate (%) and systematically analyzed by the rank-sum test. The differences were of statistical significance when *P* < 0.05.

## 3 Results

### 3.1 BMI and weight of NCP patients

As clearly shown in Table 1, compared to the profiles at admission, the BMI and weight of NCP patients increased at the time of discharge, while the differences were of no significant difference (*P* > 0.05). Only one patient was characterized by nutritional risk at admission, while all patients were free of nutritional risk at the time of discharge.

**Table 1:** BMI, weight, and NRS2002 results of NCP patients

Parameters	At admission	At the time of discharge	*t*	*P*
BMI (kg/m^2^)	24.43 ± 3.19	24.83 ± 3.05	−0.480	0.633
Weight (kg)	64.71 ± 10.53	65.75 ± 10.12	−0.375	0.709
NRS2002 ≥3(*n*)	1(3.70%)	0		0.500

### 3.2 Serum albumin and hemoglobin levels

Serum albumin synthesized by hepatic cells is predominant in serum proteins, which serve as an indicator to evaluate the nutritional status of patients. Hemoglobin refers to a protein that transports oxygen in the organism and is commonly used to evaluate the nutritional status. The serum albumin and hemoglobin levels of NCP patients were significantly elevated at the time of discharge when compared with those at admission and one week after admission (*P* < 0.05) (Table 2).

**Table 2:** Serum albumin and hemoglobin levels of NCP patients

Parameters	At admission	One week after admission	At the time of discharge	*F*	*P*
Serum albumin (g/L)	38. 24 ± 2.49	37.42 ± 3.29	40.28 ± 2.88	7.206	0.001
Hemoglobin (g/L)	128.04 ± 12.99	131.36 ± 12.66	136.54 ± 11.80	3.293	0.042

## 4 Discussion

Previous reports indicated that other visceral organ damages characterize a large number of NCP patients except for respiratory symptoms, such as severe vomiting, diarrhea, acute kidney injury, etc. [23]. Combined with the superimposed effect of Lopinavir/Ritonavir (antivirus drugs) on digestive damage, the nutrition intervention turns out to be particularly important during the treatment of NCP patients when the additive effects of antivirus drugs (like lopinavir or ritonavir), which cause alimentary tract injury, are involved. As a designated hospital for receiving and curing NCP patients, the professional team comprising specialists from the Nutrition Department of Ningbo First Hospital was soon organized to formulate nutritional recipes.

Nurses can be ideal candidates to initiate nutrition intervention procedures as they are involved in an environment filled with NCP patients. The nursing staff evaluated the weight and height profiles and the NRS-2002 scores of NCP patients. In the case of NRS-2202 score >3 for the patients with nutritional risk, the supervising physician would be informed to further assess the nutritional status. Instead, a routine diet for nutrition intervention was conducted for the patients with NRS-2002 scores lower than 3. Propaganda and education for the patients regarding diet management were carried out to highlight the importance of disease rehabilitation. As shown in Table 1, the weight of all involved NCP patients during hospitalization increased after nutrition intervention. Typically, the BMI of one patient increased from 17.99 kg/m^2^ at admission to 18.69 kg/m^2^ after 2-week nutrition intervention.

For patients with a lower BMI at admission, protein intake was increased to finally afford a weight increase of 2 kg. The intake of high-quality protein was also reported to decrease patients’ mortality rate, despite the fact that the optimal amount remains ambiguous [24,25]. Note that obesity would increase the risk of infecting NCP and affect the immune response toward virus and inflammation as well as metabolism. The reason can be that obesity alters the pathogenesis of acute respiratory distress syndrome and pneumonia [26,27]. In this work, the BMI values for patients at admission or discharge were indicative of overweight, which should be considered when a diet recipe was formulated to avoid obesity.

In the absence of approved specific therapy methods for NCP patients, immunity enhancement and symptomatic and supportive treatments constitute the main strategies. The Academy of Nutrition and Dietetics of America also recommends nutritional therapy as a routine treatment for HIV-infected patients [28]. Albumin and hemoglobin levels are closely related to nutritional status [29,30]. This study results show that nutrition intervention, especially protein intake, can significantly increase the hemoglobin and serum albumin levels of NCP patients at discharge compared with those at admission and one week after admission. The differences were of statistical significance (*P* < 0.05). In the case when the NCP patients failed to consume the food the previous day, the recipe was adjusted to better match the personal taste of the patients. For the patients with gastrointestinal discomfort, snacks (preserved plum, candies, etc.) were prepared as a complement for the use of omeprazole to enhance their compliance with the diet. The mental communication with patients helped to alleviate their psychological burden and relieve the symptoms. All these efforts finally contributed to the intake of foods formulated in the recipe.

## 5 Conclusion

In conclusion, nutrition intervention can improve nutritional status and enhance the immunity of NCP patients, thereby alleviating the symptoms and improving prognosis. However, the small sample size and simple nutritional evaluation index in this work necessitate further research with expanded samples and multiple indexes, which are anticipated to provide a theoretical basis for precise nutrition intervention in NCP patients by a multicenter study.
